# Prior test experience confounds longitudinal tracking of adolescent cognitive and motor development

**DOI:** 10.1186/s12874-022-01606-9

**Published:** 2022-06-24

**Authors:** Edith V. Sullivan, Wesley K. Thompson, Ty Brumback, Devin Prouty, Susan F. Tapert, Sandra A. Brown, Michael D. De Bellis, Kate B. Nooner, Fiona C. Baker, Ian M. Colrain, Duncan B. Clark, Bonnie J. Nagel, Kilian M. Pohl, Adolf Pfefferbaum

**Affiliations:** 1 Department of Psychiatry & Behavioral Sciences, Stanford University School of Medicine (MC5723), 401 Quarry Road, Stanford, CA 94305-5723 USA; 2 Division of Biostatistics and Dept of Radiology, University of California, San Diego, La Jolla, CA USA; 3 Department of Psychological Sciences, Northern Kentucky University, Highland Heights, KY USA; 4 Center for Health Sciences, SRI International, Menlo Park, CA USA; 5 Department of Psychiatry, University of California, San Diego, La Jolla, CA USA; 6 Department of Psychiatry & Behavioral Sciences, Duke University School of Medicine, Durham, NC USA; 7 Department of Psychology, University of North Carolina Wilmington, Wilmington, NC USA; 8 Department of Psychiatry, University of Pittsburgh, Pittsburgh, PA USA; 9 Departments of Psychiatry and Behavioral Neuroscience, Oregon Health & Sciences University, Portland, OR USA

**Keywords:** Longitudinal, Practice effects, Development, Cognition, Motor

## Abstract

**Background:**

Accurate measurement of trajectories in longitudinal studies, considered the gold standard method for tracking functional growth during adolescence, decline in aging, and change after head injury, is subject to confounding by testing experience.

**Methods:**

We measured change in cognitive and motor abilities over four test sessions (baseline and three annual assessments) in 154 male and 165 female participants (baseline age 12–21 years) from the National Consortium on Alcohol and NeuroDevelopment in Adolescence (NCANDA) study. At each of the four test sessions, these participants were given a test battery using computerized administration and traditional pencil and paper tests that yielded accuracy and speed measures for multiple component cognitive (Abstraction, Attention, Emotion, Episodic memory, Working memory, and General Ability) and motor (Ataxia and Speed) functions. The analysis aim was to dissociate neurodevelopment from testing experience by using an adaptation of the twice-minus-once tested method, which calculated the difference between longitudinal change (comprising developmental plus practice effects) and practice-free initial cross-sectional performance for each consecutive pairs of test sessions. Accordingly, the first set of analyses quantified the effects of *learning* (i.e., prior test experience) on accuracy and after speed domain scores. Then *developmental* effects were  determined for each domain for accuracy and speed having removed the measured learning effects.

**Results:**

The greatest gains in performance occurred between the first and second sessions, especially in younger participants, regardless of sex, but practice gains continued to accrue thereafter for several functions. For all 8 accuracy composite scores, the developmental effect after accounting for learning was significant across age and was adequately described by linear fits. The learning-adjusted developmental effects for speed were adequately described by linear fits for Abstraction, Emotion, Episodic Memory, General Ability, and Motor scores, although a nonlinear fit was better for Attention, Working Memory, and Average Speed scores.

**Conclusion:**

Thus, what appeared as accelerated cognitive and motor development was, in most cases, attributable to learning. Recognition of the substantial influence of prior testing experience is critical for accurate characterization of normal development and for developing norms for clinical neuropsychological investigations of conditions affecting the brain.

## Background

Longitudinal studies are considered the gold standard protocol for tracking developmental and involutional changes. By nature, longitudinal assessment requires repeated examination, ideally employing the same procedures and test materials throughout the study [1, 2]. Some assessment classes are relatively robust to repeated testing, such as measurement using structural brain imaging with MRI, somatic size, or blood chemistry panels. Even such practice-free retesting is subject to measurement drift, which can be estimated with longitudinally acquired, control data to be used as correction factors (e.g., [3, 4]). By contrast, longitudinal cognitive assessment has the intrinsic problem of *prior test experience* [5–7], also considered “practice” or “learning” [8], even when the retest interval spans one [9] to two [10–13] years. Thus, any longitudinal study purporting to track, quantify, and infer cognitive change as development, maturation, or decline is confounded by prior testing experience that requires quantification (review, [14]).

Many studies that have considered practice effects have focused on adult aging to senescence [1, 8, 15] or on repeated testing necessary in clinical settings to track the progression of CNS injury due to accident, stroke, or dementia [16–19] or recovery with treatment [20] or time [21]. Indeed, practice effects have been speculated to minimize age-related declines in older people [22, 23] and have proved useful in predicting cognitive decline or stability in patients with amnestic Mild Cognitive Impairment (aMCI). Specifically, patients with aMCI, whose cognitive scores improved with repeated testing separated by one week, showed a relatively stable disease course over one year, whereas those who showed minimal improvement between the weekly testing evidenced substantial decline over one year [24]. Thus as further emphasized by Duff and colleagues in the title of their paper [8], practice effects can be considered a “unique cognitive variable”.

A growing number of large-scale, longitudinal studies have been initiated to measure cognitive, motor, and emotional development from later childhood through young adulthood (reviewed in [25]). Among them, National Consortium on Alcohol and NeuroDevelopment in Adolescence (NCANDA) [26] with its cohort sequential design (described below) is uniquely positioned to measure practice effects and to dissociate them from developmental trajectories, modeling of which is the intent of all of these projects.

For decades, sex has been recognized as a significant moderator variable in studies of development of cognitive and motor processes (reviewed in [27, 28]). As we noted in our earlier paper [29], sex differences are associated with neuropsychological test performance during normal development and require consideration when assessing developmental trajectories of cognitive and motor functions (e.g., [30–32]). Typically, girls undergo sexual maturity earlier than boys (e.g., [33, 34]) and advance earlier than boys in language skills [35], use of semantic knowledge [36], facial emotion recognition and discrimination [37, 38], and components of episodic memory [37, 39]. By contrast, boys develop earlier than girls in mental rotation appreciation [40, 41], fine motor control (but see[42]) [43, 44], and physical strength (e.g., [45, 46]). Many sex-related differences identified are relevant to the tests used in the current study, girls tend to develop language skills earlier than boys, whereas boys develop spatial skills earlier than girls [37]. Into adolescence, as groups, girls excel on tests of memory and social cognition, whereas boys excel on tests of spatial processing and motor speed (reviewed in [27]).

Several methods have been proposed to preclude or minimize learning effects in longitudinal studies of cognitive and motor performance or, alternatively, to dissociate testing-experience learning from development. As summarized by Salthouse [5, 6] and McArdle [2], some methods, other than simply using cross-sectional protocols, include use of different test forms, staggering baseline testing, and the “twice-minus-once-tested” method [1, 5, 47]. The last approach has proved useful in studies employing an *accelerated longitudinal* design, also known as a *cohort sequential* design. To track adolescent brain, cognitive, and emotional development, the NCANDA study employed this design by initially recruiting youth in three age bands (12–14 years, 15–17 years, and 18–21 years) for subsequent annual testing [26]. The cognitive and motor functions assessed include executive functions, component processes of memory, social cognition, psychomotor speed, and visuospatial skills. Because the mainstay of the test battery is based on the computer-driven Web CNB (Computerized Neuropsychological Battery, [48, 49]), performance profiles of most of these component processes are measured in terms of accuracy, speed, and processing efficiency (the sum of standardized accuracy and speed scores) [50]. Thus, this battery assesses multiple cognitive domains, each of which can be subject to selective practice effects [2, 51].

Application of the twice-minus-once-tested method requires measurement of cross-sectional performance, for which the initial testing in NCANDA spanned ages 12 to 21 years [29], and longitudinal performance, which was measured annually using the same procedures and test materials [9]. The cross-sectional performance provided the expected developmental effect free of prior experience over a decade of adolescent growth, and the longitudinal performance included the developmental effect plus learning. The difference between the second test score of an individual (twice tested) and the first test scores of the group at the individual’s second test age (once tested) yielded an index of learning. This method revealed different extents of practice effects for the various test composites (29% to 99% of the variance was due to prior testing) and for different ages, where the younger participants showed the greatest improvement with little contribution from sex, ethnicity, or parental education [9].

The current analysis expanded the twice-minus-once-tested method to track development independent of learning from prior test experience in the NCANDA cohort over the first four years of the study. Recently, we used this approach to discriminate learning from development in the Stroop Match-to-Sample test [52], which assesses attentional inhibition, a function considered to advance over adolescence. Results indicated that learning contributed a greater proportion of the change variance than did development, which accounted for learning based prior testing [53]. The current analysis examined multiple component cognitive (Abstraction, Attention, Emotion, Episodic memory, Working memory, and General Ability) and motor (Ataxia and Speed) functions over the first four annual NCANDA test sessions. Accordingly, the first set of analyses quantified the effects of *learning* (i.e., prior test experience) on accuracy domain scores, pursuing three aims: 1) given previous longitudinal findings indicating significant learning (higher accuracy scores with prior experience) from initial repeated testing even with a year interval, we tested whether the amount and trajectory of learning between the second and later tests differed from those observed between the initial test pairs; 2) we questioned whether these parameters differed by functional domain; and 3) age and sex were examined as moderating factors. The same three aims were also applied to the speed measures with the expectation that improvement would be in the direction of faster response times; to put all accuracy and speed measures in the same direction, response speed was inverted so that larger values indicated faster performance. After quantifying learning effects, *developmental* effects were then determined for each domain for accuracy and speed, having removed the measured learning effects. We tested the hypothesis that the trajectories of scores would be different depending on the inclusion or removal of estimated practice effects, and that these trajectory differences would be present for accuracy and speed scores.

## Methods

### Participants

All participants were drawn from the NCANDA cohort of 692 who endorsed no or low levels of drinking (no-to-low alcohol drinkers) at baseline. The current longitudinal analysis required that each participant had 4 consecutive annual test sessions, starting from baseline and remained a no-to-low drinker (described below) for all included sessions. The resulting sample comprised 319 participants (154 male, 165 female), although not all participants had all composites; demographic descriptions are presented in Table 1.

**Table 1:**
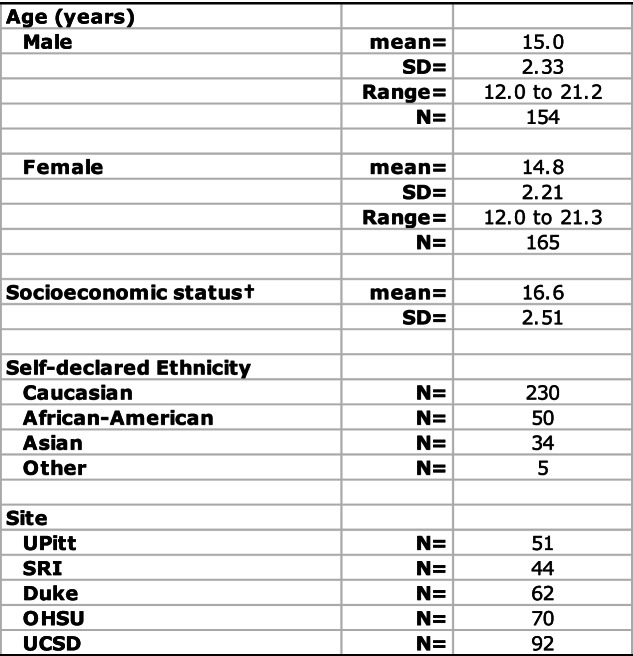
NCANDA demographics at baseline

†Highest education of a parent

All participants underwent informed consent processes at each visit with a research associate trained in human subject research protocols. Adult participants or the parents of minor participants provided written informed consent before starting the study; minor participants provided assent. The Institutional Review Boards of each site approved this study, and all methods were performed in accordance with the relevant guideline and regulations noted and approved.

### Alcohol history and testing

Participants completed the Customary Drinking and Drug use Record (CDDR, [54]) to characterize past and current alcohol and substance use. At each test session, alcohol and drug use reports were accompanied by 12-panel urine toxicology screens for amphetamine, methamphetamine, cocaine, phencyclidine, benzodiazepines, barbiturates, opiates, oxycodone, propoxyphene, methadone, tricyclic antidepressants, marijuana, and a breathalyzer for alcohol to confirm absence of evidence for recent use of drugs of abuse. Positive screens were sent for gas chromatography/mass spectrometry confirmation; if confirmed, participants were excluded from testing that day and from the current analysis.

To be considered a no-to-low drinker, participants met two sets of criteria determined with the CDDR described previously [55] as follows: 1) The *maximum lifetime drinking days* for male and female participants was ≤ 5 for age 12 to 15.9 years, ≤ 11 for age 16 to 16.9 years, ≤ 23 for age 17 to 17.9 years, and ≤ 51 for age 18 years old and older; and 2) The *maximum allowable drinks per occasion* was ≤ 3 for female participants at any age but varied by age for male participants: ≤ 3 for age 12 to 13.9 years, ≤ 4 for age 14 to 19.9 years, and ≤ 5 for age 20 years old and older.

### Cognitive and motor tests and composite score construction

Assessment was the same across all five sites and used a combination of computerized tests (originally the Web CNB, now the WebCNP (https://webcnp.med.upenn.edu/) [37, 48]) and traditional neuropsychological tests [29]. Testing was conducted by research assistants trained with annual reliability evaluations to criterion and calibrated annually by a centrally-trained psychometrician using procedures established by the NCANDA Data Analysis Resource. The tests were administered in the same order across all sites and were generally completed in approximately 3 h. Test results were uploaded to the software platform, Scalable Informatics for Biomedical Imaging Studies [56, 57] at SRI International. The longitudinal data used herein were available through a formal, locked data release (NCANDA_PUBLIC_3Y_REDCAP_V02).

The WebCNP has established construct validity and reliability and was standardized on upwards of 10,000 participants (depending on the measure) with a broad, age range (8–90 years old) [48]. Descriptions of the 15 WebCNP tests used were provided in our earlier report [29] (Supplemental Table 3 in Sullivan et al. 2016), with most tests having both accuracy and speed (response time) measures. A subset of measures from these tests was used to create theoretically-driven composite Z-scores for 8 accuracy measures (Abstraction, Attention, Emotion, Episodic Memory, Working Memory, General Ability, Balance, and Total) and 8 speed measures (Abstraction, Attention, Emotion, Episodic Memory, Working Memory, General Ability, Motor, and Total). In addition, an Efficiency score was calculated as the sum of the Total Accuracy plus Speed Z-scores [50]. The individual tests and computed composites were described previously, where Table 2 lists the cognitive and motor domains and specific processes assessed, with associated brain regions reported to support each process (see Supplemental table 2 in Sullivan et al. [29] also lists the composite domains, test measures and variable names entered into each composite domain, and scoring procedure for each measure).

Composite score construction followed three steps [37, 58]. First, each measure was standardized on baseline scores achieved by all male and female adolescents who met NCANDA entry criteria (maximum *N* = 319) and expressed as a Z-score (mean = 0 ± 1SD). This transformation function was applied to all subjects at all times. Not all participants had scores for all measures, typically due to computer failure, participant’s refusal to perform a test, or lack of testing time; the number of participants with scores are in Table 2 in the Results. Next, all scores for which a low score signified good performance were transformed by multiplying scores by -1 so that high scores for all measures were in the direction of good performance. Finally, the mean Z-score of all individual measures that comprised a composite was calculated; missing scores were allowed, but each composite score had to have at least 2 measures to make a domain.

### Statistical analysis

The primary analysis tools were the General Additive Mixed Model (GAMM) and Likelihood Ratio Tests (LRT) using the *gamm* and *anova* functions from the *mgcv* package in R Version 3.1.0 [http://www.r-project.org]. Age was allowed to be a nonlinear smooth effect, implemented via thin plate splines [s(age)] with 3 internal knots [59], herein after referred to as “smoothed age.” Roughness penalties for the smooth effects were estimated using generalized cross validation [60].

#### Estimation of learning from visit to visit

To determine the learning effect from visit to visit, three data sets were constructed: 1) all subjects’ visit 1 plus visit 2; 2) all subjects’ visit 2 plus visit 3; 3) all subjects’ visit 3 plus visit 4. For each dataset, a regression analysis fitting the data with age (GAMM with smoothed age) was performed without and with visit as a factor. The results of the two models were compared with an LRT; significant improvement by adding visit to the model indicated significant learning from visit to the subsequent visit. The improvement in the amount of variance explained (R-squared) is reported here as an index of the amount of learning between visits.

To test for age effects on learning, for each test visit pair, GAMMs with and without an age-by-visit interaction were compared using LRTs. A significantly better fit with age-by-visit interaction indicated significant age effects on learning.

To test for sex effects on learning, for each visit pair, GAMMs with age-by-visit plus sex-by-visit were performed and examined for learning-by-sex interactions.

#### Learning-adjusted developmental model

To quantify learning across the four visits, a sequence of model fits was performed [53] that allowed the estimation of development effects independent of learning effects. The learning-adjusted development estimate across sessions was calculated as follows:

The *cross-sectional fit* of dependent variable y vs. age was computed across all participants for each visit separately producing: *fit1 (based on only 1*^*st*^* visits), fit2 (based on only 2*^*nd*^ *visits), fit3 (based on only 3*^*rd*^* visits), fit4 (based on only 4*^*th*^* visits).*

For visits 2, 3, and 4, the *age-related learning effect* from the previous visit was estimated by computing the difference between the predicted values from the cross-sectional fit at the current visit minus the predicted value when applying the fit from the previous visit to the ages at the current visit. This procedure was done cumulatively across visits 2, 3, and 4, producing learning-adjusted (i.e., learning-removed) values. Because visit 1 had no learning relevant to these test sessions, visit 1 values were not adjusted for experience effects. This adjustment is a direct extension of the “once vs. twice tested” method to more than two testing occasions.

The estimated age-dependent learning at visit 2 was the difference between the predicted values of cross-sectional ***fit2*** applied to subject visit 2 ages minus the predicted values of cross-sectional ***fit1*** applied to the same visit 2 ages:

$$visit2.adj=visit2-(predict(fit2\_on\_visit2)-predict(fit1\_on\_visit2))$$

This is the simple case of baseline with one follow-up test session as used in Sullivan et al. [9] and is the “once minus twice tested” method [6].

For subsequent visits the learning effect required testing of additional learning from visit to visit, calculated as follows: The estimate of learning at visit 3 was the difference between the predicted values of cross-sectional ***fit3*** applied to subject ages at visit 3 minus the predicted values of cross-sectional ***fit2*** again applied to subject ages at visit 3. This quantity was then added to the estimate from visit 1 to visit 2 to obtain the cumulative learning effect from baseline to visit 3:

$$\begin{array}{c}visit3.adj=visit3-[(predict(fit2\_on\_visit2)-predict(fit1\_on\_visit2))\\ +\\ (predict(fit3\_on\_visit3)\boldsymbol{ }-predict(fit2\_on\_visit3))]\end{array}$$

The estimate of cumulative learning at visit 4 was the difference between the predicted values of cross-sectional ***fit4*** applied to subject ages at visit 4 minus the predicted values of cross-sectional ***fit3*** applied to the same subject ages at visit 4, which was then added to the estimate from visit 1 to visit 2 and from visit 2 to visit 3:

$$\begin{array}{c}visit4.adj=visit4-[(predict(fit2\_on\_visit2)-predict(fit1\_on\_visit2))\\ +\\ \begin{array}{c}(predict(fit3\_on\_visit3) -predict(fit2\_on\_visit3))\\ +\\ (predict(fit4\_on\_visit4)-predict(fit3\_on\_visit4))]\end{array}\end{array}$$

To examine the effect of age on performance, GAMMs examining composite scores as a function of smoothed age were performed before and after adjusting for learning. ANOVAs were computed to allow comparison of the GAMM with smoothed age to the GAMM with linear age to determine whether the developmental trajectory of that test composite was better describes as smoothed or linear.

To test for sex effects on development across all visits, GAMMs examining learning-adjusted values as a function of smoothed and linear age with and without sex as a factor were compared with ANOVA.

To account for the multiple comparisons made, family-wise Bonferroni correction was determined for 8 test session pairs for each metric (accuracy and speed) with α = 0.05 required *p*-values ≤ 0.00625 (two-tailed) to be considered significant.

## Results

The first set of results quantifies the learning effects for each performance metric (accuracy and speed) of each composite score by age and sex. The second set quantifies the developmental effects with the measured learning (i.e., practice) effects removed.

### Learning effects

**Fig. 1:**
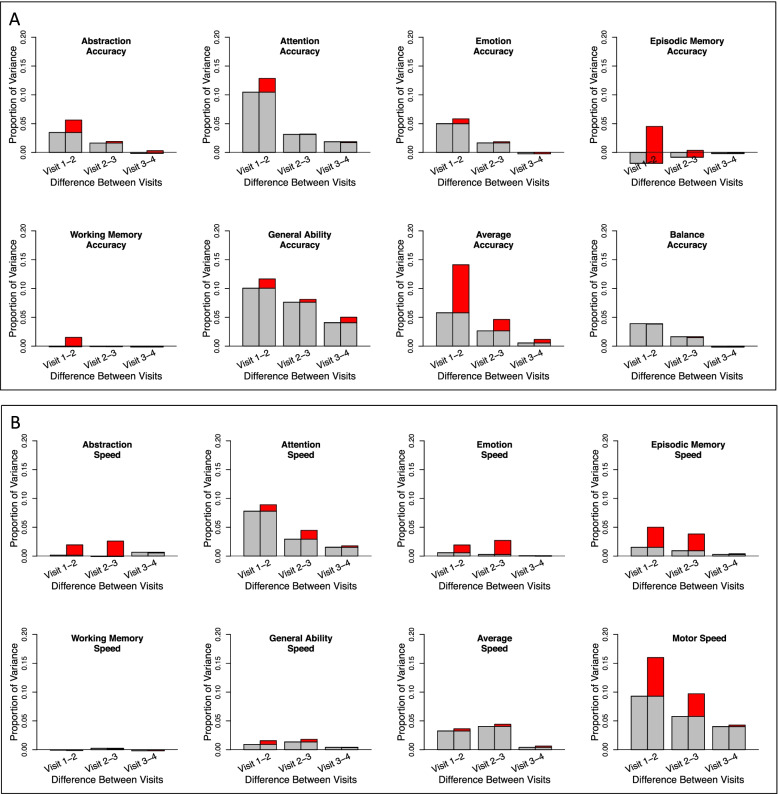
The difference in variance explained by age between each visit pair (e.g., visit 1 compared to visit 2) with age alone in the left bar of each pair and age + visit in the right bar of each pair, with the additional variance explained by learning depicted in red in the right bar of each visit pair

**Fig. 2:**
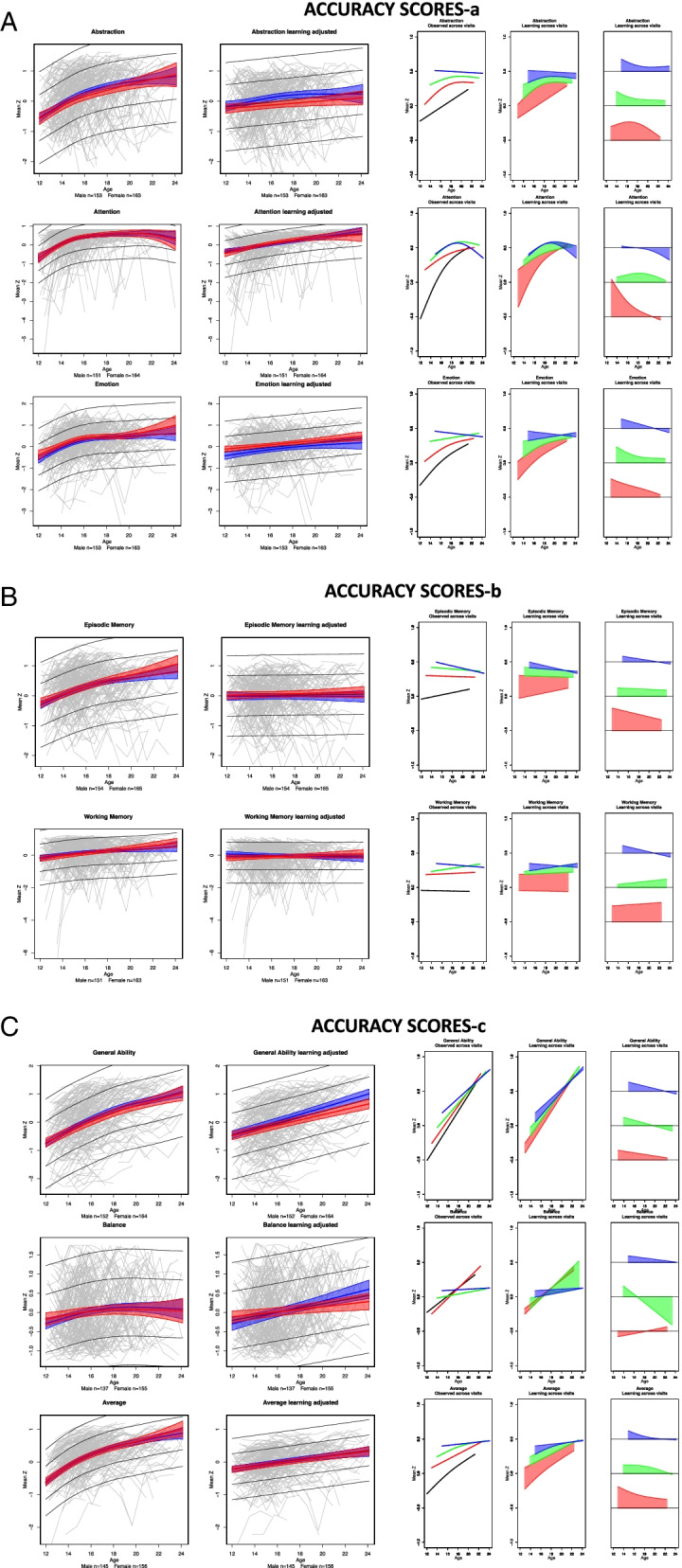
**a-c** Two left panels: The gray spaghetti plots show accuracy performance of each person for each of the four test sessions for each test composite. The gray regression lines indicate the ± 1 and ± 2 standard deviations of all participants. The color regression lines indicate the mean and 95% confidence interval of the performance by male (blue) and female (red) participants. The left plots show the learning + developmental effect; the right plots show the learning-adjusted developmental effect. Three right panels depict learning by session in accuracy scores. The first plot presents the fit of the cross-sectional scores at each test session over age: black = test 1, red = test 2, green = test3, and blue = test 4. The second plot displays the learning between tests 1–2 (red), tests 2–3 (green), and tests 3–4 (blue) over age. The third plot also displays the learning over age between test pairs normalized at 0 to reveal age effects and their differences between test pairs. The general trend was for the younger participants to show greater learning than the older ones especially between sessions 1 and 2 (red filled plots)

**Fig. 3:**
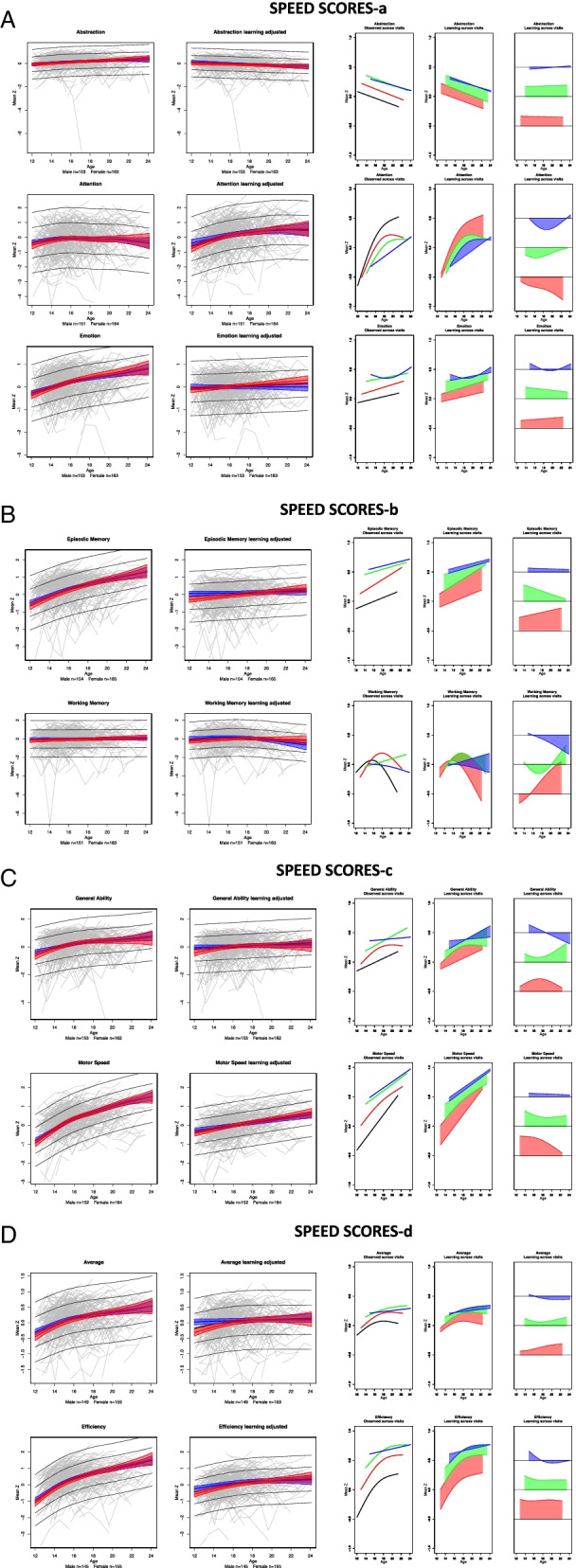
**a-d** Two left panels: The gray spaghetti plots show speed performance of each person for each of the four test sessions for each test composite. The gray regression lines indicate the ± 1 and ± 2 standard deviations of all participants. The color regression lines indicate the mean and 95% confidence interval of the performance by male (blue) and female (red) participants. The left plots show the learning + developmental effect; the right plots show the learning-adjusted developmental effect. Three right panels depict learning by session in speed scores. The first plot presents the fit of the cross-sectional scores at each test session over age: black = test 1, red = test 2, green = test3, and blue = test 4. The second plot displays the learning between tests 1–2 (red), tests 2–3 (green), and tests 3–4 (blue) over age. The third plot also displays the learning over age between test pairs normalized at 0 to reveal age effects and their differences between test pairs. Unlike the accuracy scores, the general trend for the speed scores showed different age trends for the different test composites

For each composite score, learning was quantified by computing the difference in the variance explained by age between pairs of two GAMM models with and without visit as a factor; these statistics are presented in Table 2 along with the percent change (typically improvement), their associated LRT L ratios of the model fits, and *p*-values. The additional variance explained by age plus visit in the model is indicative of learning and is depicted in red in the second bar of each visit pair in Fig. 1. The learning effect did not differ significantly by sex between test session pairs for any accuracy composite score but showed a modest sex effect for the Motor speed composite, described below (Table 3). The trend was for the younger participants to show greater learning than the older ones especially between sessions 1 and 2 (Figs. 2 and 3, column 5). Cross-sectional scores at each test session *over age* are presented in Figs. 2 and 3 (first of the 3 right panels), and the learning component is presented in the second and third figures of the right triplet for each composite score over age.

**Table 2:**
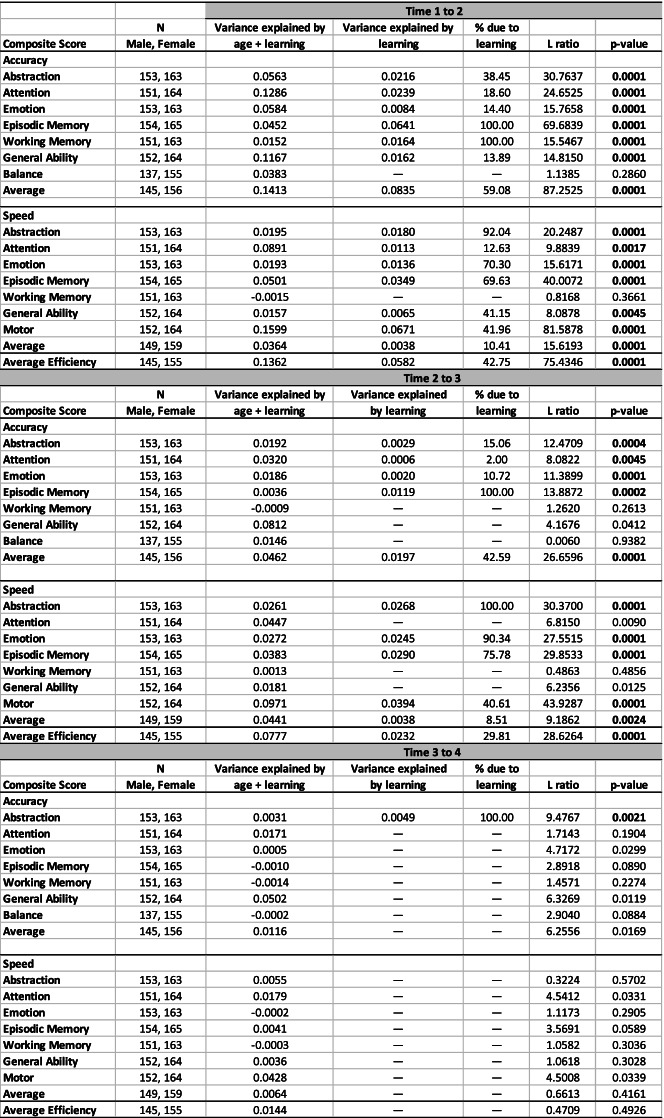
R2 for each test session gamm and difference between test session pairs tested with ANOVA indicative of learning

†Improvement in R2 between a pair of test sessions; see red values in Fig. 1 bar plots

Bold values are significant with a family-wise Bonferroni correction for 8 comparisons (alpha = 0.05) at *p* ≤ 0.00625

% due to learning values are noted only for significant improvement

**Table 3:**
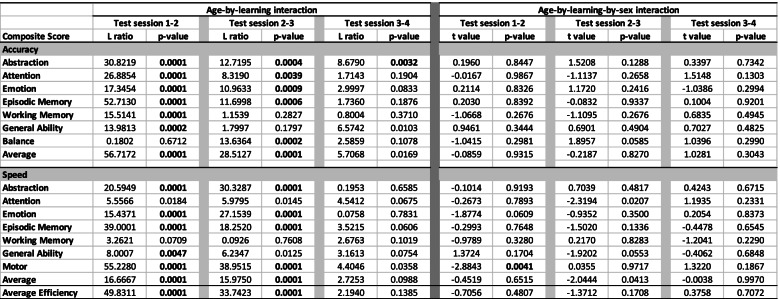
Interactions of age or sex with learning between test session pairs

NB: See left panel of spaghetti plots for learning + development

Bold values are significant with a family-wise Bonferroni correction for 8 comparisons (alpha = 0.05) at t *p* ≤ 0.00625

#### Accuracy composite scores

Overall, smooth age fits were better than linear age fits in describing the unadjusted data, which comprised both learning and development (Table 4; Fig. 2a-c, left spaghetti plots for each composite). Two exceptions were Working Memory and Balance; the latter showed a smooth trend.

**Table 4:**
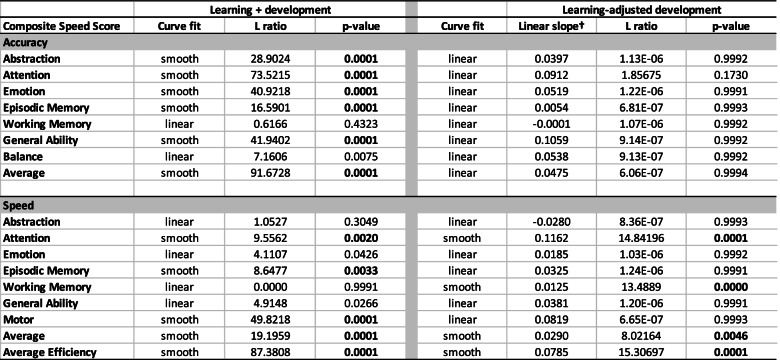
Test for linear vs. smooth fit across all sessions (with sex in the model) for development with and without learning effects

Bold values are significant with a family-wise Bonferroni correction for 8 comparisons (alpha = 0.05) at *p* ≤ 0.00625

See Figs. 2 and 3 spaghetti plots: left panel = learning + development; right plots = learning-adjusted development

†Slopes are taken from linear models and estimate the Z-unit change per year

For *Abstraction* accuracy, the ANOVA comparing the GAMM fits between each successive pairs of test sessions indicated significant performance improvement between each pair (red area of Fig. 1 and Table 2). In all three cases, significant age-by-learning interactions indicated that learning was greater with younger age.

For *Attention, Emotion*, *Episodic Memory* and *Average* accuracy, the ANOVA comparing the GAMM fit pairs of test sessions indicated significant improvement from time 1 to 2 and 2 to 3 but not from 3 to 4. Further, learning interacted with age, indicating greater learning with younger age. For *Working Memory* and *General Ability* accuracy, the ANOVA revealed significant improvement that interacted with age from time 1 to 2. *Balance* was the only composite failing to show significant improvement between any test pairs and no interaction with age.

#### Speed composite scores

Like Accuracy, Speed showed improvement over test sessions, but the overall pattern of improvement in composite scores differed by metric. Smooth age fits were better than linear age fits in describing the unadjusted data, which comprised both learning and development (Table 4; Fig. 3a-d, left spaghetti plots for each composite), for four of the eight test composites and the Efficiency score: Attention, Episodic Memory, Motor, and Average Speed. Linear fits better described the age effect for the four remaining composites: Abstraction, Emotion, Working Memory, and General Ability; Emotion and General Ability showed smooth trends.

The ANOVAs comparing the GAMM fits revealed significant increases in speeded responses between the first two pairs of test sessions but not the last pair for five composite scores and for Average Efficiency: Abstraction, Emotion, Episodic Memory, Motor, and Average Speed. Attention and General Ability speed improved from time 1–2 only. Working Memory showed no improvement between any test pair (Fig. 3a-d, 3 plots in the right panel; Table 2).

The age-learning interaction was significant for time 1–2 and time 2–3 for Abstraction, Emotion, Episodic Memory, Motor, and Average Speed (Table 3, Fig. 3a-d). For General Ability speed, the interaction with age was significant between time 1–2 and showed a trend between time 2–3.

The learning effect in the speed scores between test session pairs differed by sex for the Motor composite only. The sex difference occurred between tests 1 and 2 and indicated that the female participants showed a greater gain in speed than the male participants (see differences in confidence intervals for female scores in red relative to male scores in blue in Fig. 3, left panel).

### Learning-adjusted developmental effects

#### Accuracy composite scores

These results describe developmental effects for each composite after removing the estimated learning effects. For all 8 composite accuracy scores, the developmental effect was significant across age and was adequately described by linear fits with no improvement from nonlinear (smooth) fits (*p* = 0.999 for all composites except Attention *p* = 0.173) (Table 4; Fig. 2, right panel of spaghetti plots). Thus, the accelerated improvement in scores over age (Fig. 2 spaghetti plots in left panels) was attributed to greater learning rather than apparent accelerated development in the younger relative to the older participants.

#### Speed composite scores

The learning-adjusted developmental effects for speed were adequately described by linear fits with no improvement from smooth fits (*p* = 0.999) for *Abstraction, Emotion, Episodic Memory, General Ability,* and *Motor* scores. The smooth fit was better than the linear fit for *Attention, Working Memory, Average Speed*, and *Efficiency* (Table 4; Fig. 3 right spaghetti plots).

**Fig. 4:**
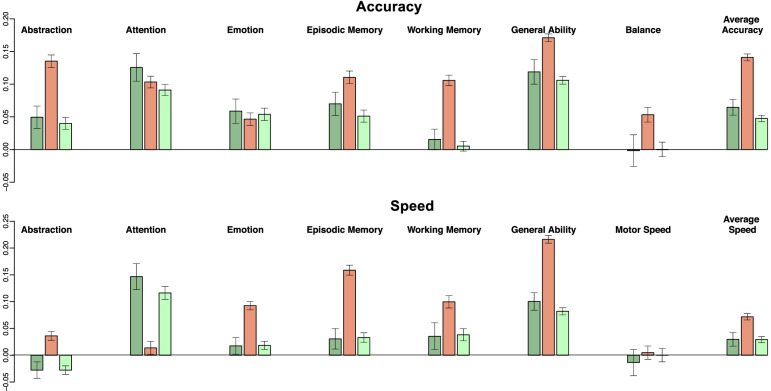
Top: Accuracy; bottom: Speed. Dark green = rate of change/year from cross-sectional analysis of baseline data. Salmon = rate of change/year from fixed effects of mixed-model analysis of data across all 4 years. Light green = rate of change/year from fixed effects of mixed-model analysis of learning-adjusted data across all 4 years

The effect of learning adjustment can also be portrayed by comparing the cross-sectional age relation to the longitudinal age relation with and without learning adjustment as per Salthouse [61]. Figure 4 presents the average slope from the simple cross-sectional linear regression at baseline (value in Z units/year) compared to the fixed effects from a linear mixed-model regression of the data across all 4 years before and after learning adjustment for the accuracy and speed domains. With few exceptions (notably, Attention speed; but even in this instance, the learning-adjusted better reflected the cross-sectional results than ignoring the practice), the non-adjusted data overestimated the rate of change per year and the learning adjusted more closely reflected the initial cross-section age relation.

## Discussion

In general, mean scores for both accuracy and speed improved on most composite scores across the four test sessions with the greatest gains between the first and second tests (Fig. 2, two right panels). Abstraction was the only composite to show improvement over all 3 comparisons. Except for Working Memory, the overall learning for the Accuracy composite scores was greater in younger than older participants (Fig. 2, column 5). The Balance composite, which assessed gait and quiet standing, was unique in showing no significant change between any test session. Learning was detectable even in the context of apparent ceiling effects in the Attention and Working Memory composites (Fig. 2, spaghetti plots). Unlike the Accuracy scores, the Speed composite scores did not show greater learning in the younger than older participants, except for the Motor Speed composite.

We tested the hypothesis that the trajectories of scores would be different depending on the inclusion or removal of estimated practice effects, and that these trajectory differences would be present for accuracy and speed scores. For most composite scores, these hypotheses were supported. Specifically, developmental effects were linear for all accuracy composites. Thus, what appeared as accelerated advancement in the younger boys and girls was attributable to the learning component of the aging functions. As with the linear trends for accuracy, five learning-adjusted speed scores were linear. Speed scores showing smooth fits were Attention, Working Memory, Average Speed, and Efficiency; each exhibited a slight inflection, notably in the younger ages that were modestly steeper for the girls than the boys. In all cases, the confidence intervals of the female participants overlapped or exceeded the speed score intervals of their male counterparts, albeit non-significant from the GAMM analyses, thereby lending little support for a male advantage in speeded responding in cognitive or motor realms in later adolescence (cf. [27]). Further, sex differences may be attenuated with multiple annual test sessions, noted herein. Absence of age-by-sex interactions was also reported in a 4-year longitudinal study of youth tested every 2 years (ages 6 to 18 years at baseline testing) despite sex-related performance differences in specific tests: male youth achieved better scores than female youth on Block Design, whereas the opposite occurred on Grooved Pegboard and Digit Symbol Coding tests [11]. One interpretation is that the sex differences were stable despite repeated testing and presumed further development over the 4-year interval.

The original, cross-sectional analysis of the WebCNP composite scores noted that sex differences had smaller effect sizes than age but were evident, with female participants outperforming their male counterparts on attention, word and face memory, reasoning speed, and all social cognition tests, whereas male participants outperformed their female counterparts in spatial processing and sensorimotor and motor speed [37]. Comporting with those cross-sectional findings, our current longitudinal observations revealed that these sex differences were greatest at younger ages, with adolescent development, female participants became faster over time on Motor Speed.

To quantify practice effects associated with subtests of the computer-based test battery *Cognition,* which is based on the Web CNP, Basner and colleagues [15] varied testing parameters, including test forms and test–retest intervals for retesting upwards of 15 times. Remarkably, even their 6 subtests using unique stimuli in subsequent test sessions evidenced practice effects, consistent with the interpretation that some form of procedural learning beyond episodic memory for specific test information contributes to practice effects, that is, prior experience.

In the current study, the variance explained by age + learning in the visit-to-visit analyses ranged from less than 0 to 16%. Within those totals, the proportion of accuracy score variance attributable to *learning* ranged from 14% for Emotion to more than 100% for the two Memory composites between test sessions 1 and 2 (Fig. 1 and Table 2). For Average accuracy, the gain was nearly 60% from tests 1 to 2 and remained high between tests 2 to 3 at 42%. Learning-associated improvement in speed scores between sessions 1 and 2 was especially high for Abstraction (92%), Emotion (70%), and Episodic Memory (69%). Thus, a unique contribution of this analysis was to address whether practice effects in this adolescent to young adult age range would accrue beyond the first follow-up testing and, if so, would occur in all functional domains examined. Although the greatest learning effect occurred between the first and second visits, further learning was measurable between visits 2 and 3 and again between visits 3 and 4 for two accuracy scores (General Ability and Average) and for several speed scores and the Efficiency score, as depicted in the red segments in Fig. 1.

The slopes from the linear models describe the *learning-adjusted developmental* performance trajectories (Table 4) in terms of Z-unit changes per year (Figs. 2 and 3 right spaghetti plots). Extrapolating from the youngest to the oldest youth, the functional composites showing the largest improvement were General Ability accuracy and Attention accuracy and speed, with estimated gains of approximately 1.0 Z-unit over a decade. The developmental estimates would have been inflated had they not been adjusted for learning. Indeed, a benefit of the cohort sequential design in longitudinal assessment was demonstrated in our analysis, based on Salthouse [61], that compared cross-sectional slopes, longitudinal slopes unadjusted for practice effects, and longitudinal slopes adjusted for practice effects (Fig. 4). Use of the twice minus once tested analysis enabled this comparison, which revealed that for most composites the longitudinal slopes adjusted for practice effects reflected the cross-sectional slopes, a pattern previously noted in a longitudinal analysis of cognitive performance by men and women spanning the adult age range [1]. By contrast, the unadjusted longitudinal slopes were substantially greater and thus over-estimated the developmental trajectories. Critically, longitudinal sessions initiated at a single or narrow age preclude such an adjustment, which requires cross-sectional observations to be made over wide age bands.

### Limitations

Although use of composite scores can reduce excessive variance often observed in individual tests, the test composites created in the current study, which were similar to those used by Gur and colleagues [48–50, 62], comprised different numbers of measures that may have contributed to differences in variances. Further, some tests may be more difficult than others, and difficulty levels may differ by variables such as age, sex, or individual abilities. Despite the strength of the twice-minus-once-tested method, representation of each age in adolescence had a limited sample size, which was then halved in the sex analyses. This method may also be subject to cohort differences by recruitment age bands [1, 2, 63].

## Conclusion

Longitudinal study, held as the gold standard for tracking developmental trajectories, must take prior assessment experience, also considered learning or practice effects, into account. Study protocols that recruit all participants at one age or within a narrow age band are not positioned to use the twice-minus-once-tested method to dissociatate learning from development, whereas studies using the cohort sequential (accelerated longitudinal) design are poised to do so. Had our method not dissociated the effects of learning from development, the course of developmental changes over the adolescent years would have been interpreted as following an accelerating increase, notable in younger ages. By contrast, removing the learning effects revealed a linear developmental trajectory for all accuracy composite scores for all cognitive functions examined. Recognition of the substantial influence of prior testing experience, which does not necessarily rely on repetition and memory for specific test items (cf., [15]) and can be a metric of interest in its own right [8], is critical to be accomplished in highly vetted groups of adolescents and emerging adults. Doing so will enable accurate characterization of normal development and provide norms for other uses, including clinical neuropsychological investigations of conditions affecting the brain whatever the cause.

## Data Availability

The data were part of the public data release NCANDA_PUBLIC_3Y_REDCAP_V02*, distributed according to the NCANDA Data Distribution agreement.** * Pohl KM, Sullivan EV, Podhajsky S, Baker FC, Brown SA, Clark DB, Colrain IM, De Bellis MD, Nagel BJ, Nooner KB, Tapert SF, Pfefferbaum A: The `NCANDA_PUBLIC_3Y_REDCAP_V04` Data Release of the National Consortium on Alcohol and NeuroDevelopment in Adolescence (NCANDA), Sage Bionetworks Synapse. https://dx.doi.org/10.7303/syn26350358 **https://www.niaaa.nih.gov/ncanda-data-distribution-agreement

## Abbreviations

NCANDA: National Consortium on Alcohol and NeuroDevelopment in Adolescence
CDDR: Customary Drinking and Drug use Record
WebCNB: Web Computerized Neurocognitive Battery
WebCNP: Web Computerized Neuropsychological Testing System
GAMM: General Additive Mixed Model
LRT: Likelihood Ratio Tests
ANOVA: Analysis of variance
